# Intolerance of uncertainty and psychosis: A systematic review

**DOI:** 10.1111/bjc.12509

**Published:** 2024-10-22

**Authors:** Jayne Morriss, Daisy Butler, Lyn Ellett

**Affiliations:** ^1^ School of Psychology, Faculty of Environmental and Life Sciences University of Southampton Southampton UK

**Keywords:** delusions, hallucinations, intolerance of uncertainty, paranoia, psychosis, schizophrenia

## Abstract

**Objectives:**

Intolerance of uncertainty, the tendency to interpret and react negatively to uncertainty, is a transdiagnostic risk factor for anxiety, depression and eating‐related disorders. Given the high comorbidity between anxiety, depression and schizophrenia‐spectrum diagnoses (SSDs), there is potential for intolerance of uncertainty to play a role in modulating psychosis symptoms. To address this gap in our understanding, we conducted the first prospectively registered systematic review on intolerance of uncertainty and psychotic symptoms in both people with SSDs and in the general population.

**Methods:**

Four databases were searched (PsycINFO, Medline, Web of Science and PubMed), which identified ten studies with a total of 1503 participants that measured intolerance of uncertainty and psychosis symptoms.

**Results:**

Key findings suggest the following: (1) Intolerance of uncertainty was associated with total negative psychotic symptoms with small–medium effect sizes; (2) intolerance of uncertainty was higher in individuals with an ‘at‐risk’ mental state for psychosis compared to controls; (3) higher intolerance of uncertainty was associated with more individual psychotic symptoms related to delusions and paranoia within clinical and nonclinical samples; and (4) there was mixed evidence for a relationship between intolerance of uncertainty and auditory hallucinations and intolerance of uncertainty and total positive symptoms in clinical samples.

**Conclusions:**

Overall, these findings highlight that intolerance of uncertainty may be an important transdiagnostic dimension and potential treatment target for psychotic symptoms such as delusions and paranoia in people with SSDs.

**Practitioner points**

- Intolerance of uncertainty may be an important transdiagnostic risk factor for schizophrenia spectrum disorders.
- Intolerance of uncertainty may be an important treatment target for psychological therapies for SSDs.
- Intolerance of uncertainty may be a particularly important treatment target for individuals with delusions and paranoia.

## INTRODUCTION

Intolerance of Uncertainty “is a dispositional incapacity to endure the aversive response triggered by the perceived absence of salient, key, or sufficient information, and sustained by the associated perception of uncertainty” (Carleton, 2016, p. 31). A wealth of research has demonstrated that individuals with higher self‐reported intolerance of uncertainty, relative to lower self‐reported intolerance of uncertainty, tend to interpret (Cupid et al., 2021; Pepperdine et al., 2018) and react to uncertainty negatively (for reviews, see Morriss et al., 2021; Sahib et al., 2023; Tanovic et al., 2018). Furthermore, intolerance of uncertainty is normally distributed across the general population (for review see Carleton, 2016), and higher self‐reported intolerance of uncertainty is commonly observed in individuals presenting with anxiety (e.g. generalized anxiety, obsessive‐compulsive, post‐traumatic stress), depression and eating‐related disorders (McEvoy et al., 2019). Crucially, intolerance of uncertainty and associated symptom severity (e.g. negative affect, worry, obsessions) are reliably reduced through cognitive behavioural therapies for anxiety and depression (Miller & McGuire, 2023; Wilson et al., 2023). Given these promising results, intolerance of uncertainty is now considered a well‐established transdiagnostic risk factor and treatment target for anxiety and depression‐related disorders.

An emerging body of research on the positioning of intolerance of uncertainty within hierarchical models of transdiagnostic vulnerabilities for psychopathology (for discussion, see Morriss, 2023) has postulated intolerance of uncertainty as a core lower‐order transdiagnostic dimension underlying the higher order (latent) factor of negative affectivity (also known as neuroticism) (Carleton, 2016). Empirical research on hierarchical models of transdiagnostic vulnerabilities for anxiety and depression disorders has demonstrated that intolerance of uncertainty accounts for larger factor loadings, compared to other lower‐order transdiagnostic dimensions, in supporting negative affectivity (Hong & Cheung, 2015; Paulus et al., 2015). Notably, negative affectivity is not only limited to anxiety and depression disorders but also features in schizophrenia spectrum diagnoses (SSDs) (Ohi et al., 2016; Van Os & Jones, 2001). Thus, negative affectivity is a shared higher order factor across anxiety, depression and SSDs, which may partly explain the high comorbidity across these different mental health disorders (Braga et al., 2013). Based on this evidence, there is also potential for intolerance of uncertainty to support negative affectivity and contribute to psychotic symptoms in SSDs.

Experiences of negative affect (e.g. anxiety) and worry that are commonly associated with intolerance of uncertainty (for reviews, see Carleton, 2016; Milne et al., 2019), have also been linked to the formation and exacerbation of psychotic symptoms, including persecutory delusions and auditory hallucinations (‘hearing voices’) (Freeman et al., 2002; Freeman et al., 2012; Garety et al., 2001; Knežević et al., 2019; Ludwig et al., 2019; Sun et al., 2018). Such paranoid thinking is common in the general population (Bebbington et al., 2013; Ellett et al., 2003; Ellett, Varese, et al., 2023; Ellett, Wildschut, & Chadwick, 2023; Fenigstein & Vanable, 1992; Freeman et al., 2021; Kuipers et al., 2019), consistent with continuum models (Strauss, 1969; Elahi et al., 2017). Paranoid beliefs are posited to come about as a way of coping with uncertainty (Preti & Cella, 2010). Given this theoretical argument, it is possible that intolerance of uncertainty may interact with psychotic symptoms such as paranoia in the general population and in SSDs.

As far as we are aware, there is no existing review of empirical evidence on the relationship between intolerance of uncertainty and psychotic symptoms in the general population and those with SSDs. Addressing this question will shed light on whether intolerance of uncertainty and psychotic symptoms interact at the general population level, as well as whether intolerance of uncertainty is an important transdiagnostic risk factor for psychosis symptoms in SSDs, with potential implications for considering intolerance of uncertainty as a treatment target in such disorders. The following systematic review examined whether intolerance of uncertainty is related to psychotic symptoms in both individuals with SSDs and in the general population and addressed the following research questions: (1) What is the relationship between intolerance of uncertainty and psychotic symptoms, including positive and negative symptoms, and individual symptoms including paranoia, persecutory delusions and auditory hallucinations (‘hearing voices’)? and (2) Are there similar or different relationships between intolerance of uncertainty and psychotic symptoms in nonclinical and clinical populations?

## METHOD

**FIGURE 1:**
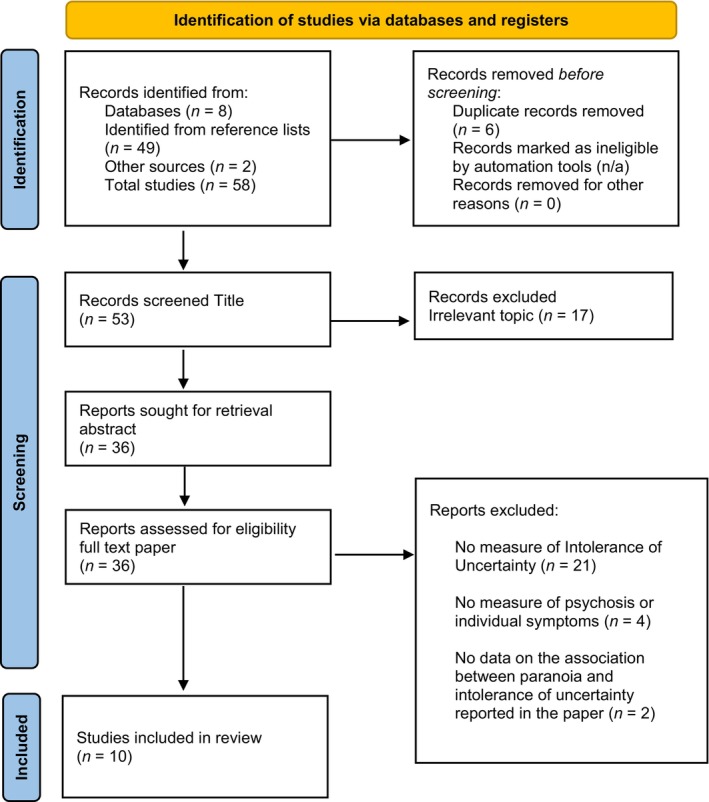
PRISMA diagram showing search process.

The review was pre‐registered on the OSF (reference: osf.io/h7gpy). The following four databases (PsycINFO, Medline, Web of Science, PubMed) were searched up to March 2024. Abstracts and titles were searched for the following: (psychosis OR schizophren* OR hallucinat* OR voices OR delusion* OR paranoi* OR positive symptoms OR negative symptoms) AND (Intolerance of Uncertainty OR uncertainty OR intolerance of ambiguity). Inclusion criteria for the review were: (1) studies that used a quantitative design; (2) assessed psychotic symptoms using a published questionnaire with good psychometric properties (e.g. reliability with Cronbach's alpha >0.7 and construct validity reported); (3) assessed intolerance of uncertainty using a published questionnaire with good psychometric properties (e.g. reliability with Cronbach's alpha >0.7 and construct validity reported); (4) clinical or nonclinical population. Exclusion criteria were: (1) qualitative studies; (2) case studies; (3) existing reviews; (4) studies with child or adolescent populations; (5) theses or dissertations. Among the studies meeting our inclusion criteria, only English‐language and peer‐reviewed articles were considered, without restrictions on the publication year. After eliminating duplicate articles, each paper was screened independently by LE and DB based on its title, abstract, or full text to assess suitability. There were no discrepancies in the assessment of the eligibility of papers by the independent raters (LE and DB). The review included seven papers, and Figure 1 summarizes the search process. Effect sizes were reported as small (*r* = .10), medium (*r =* .30) or large (*r* > .50) in studies reporting associations, and Cohen's *d* of .80 or higher was deemed large for group comparisons (Cohen, 1992). Each study was assessed for quality using the EPHPP tool (Thomas et al., 2004), and overall quality ratings by study are shown in Table 1.

**TABLE 1:** Characteristics of studies, main findings and quality assessment.

Authors	Sample	Design	Paranoia/psychosis measure	Intolerance of uncertainty measure	Main findings	Quality rating
Bredemeier et al. (2019)	Outpatient sample with psychosis (*n* = 252)	Cross‐sectional	SAPS	IUS‐9 item (8 items from original 27 items and 1 new item)	Correlation between	Weak
			PSYRATS delusions		IUS and SAPS delusions (*r* = .14)*	
			PSYRATS hallucinations		IUS and PSYRATS delusions (*r* = .20)*	
			PSYRATS distress		IUS and SAPS hallucinations (*r* = .07)	
			SANS		IUS and PSYRATS voices (*r* = .07)	
					IUS and PSYRATS distress (*r* = .18)*	
					IUS and SANS (*r* = .17)*	
Broome et al. (2007)	At‐risk mental state for psychosis (*n* = 35) and matched healthy controls (*n* = 23)	Cross‐sectional	CAARMS	IUS‐27‐item measure	At‐risk mental state	Moderate
		Between‐subjects	PDI		IUS (M = 79.8, SD = 22.8)	
			PANSS			
			SAPS: delusion subscale		Controls	
					IUS (M = 58.3, SD = 15.3)	
					Difference significant *p* < .0005	
					Cohen's *d* = 1.11, large effect size	
King and Dudley (2017)	Students (*n* = 102)	Cross‐sectional	GPTS	IUS (12 items)	IUS and GPTS (*r* = .50)**	Moderate
Larsen et al. (2021)	Students	Cross‐sectional	RGPTS	IUS (12 items)	Study 1: IUS and RGPTS (*r* = .46)**	Weak
	(*n* = 261 study 1)				Study 2: IUS and RGPTS (*r* = .38)**	
	(*n* = 258 study 2)					
Lebert et al. (2021)	Clinical population with psychosis	Cross‐sectional	PSYRATS delusion subscale	IUS Short form (2 items)	IUS and PSYRATS delusion subscale (*r* = −.05)	Weak
	(*n* = 24)		PANSS Positive Total		IUS and PANSS Positive Total (*r* = −.20)	
			PANSS item 6 on paranoia		IUS and PANSS paranoia (*r* = .72)*	
Sedighi et al. (2019)	Patients with schizophrenia	Cross‐sectional	PANSS	IUS (12 items)	IUS and positive symptoms (*r* = .53)*	Moderate
	(*n* = 60)				IUS and negative symptoms (*r* = .66)*	
Startup et al. (2016)	Patients with persecutory delusions (*n* = 150)	Cross‐sectional	GPTS	IUS‐27‐item measure	IUS and GPTS (*r* = .48)**	Moderate
			PSYRATS delusions		IUS and PSYRATS delusions (*r* = .39)**	
White and Gumley (2010)	Clinical population with psychosis (*n* = 27)	Cross‐sectional	BAPS (short form) 4 subscales: negative beliefs about paranoia, beliefs about paranoia as a survival strategy, general positive beliefs, normalizing beliefs.	IUS‐27	IUS with BAPS: negative beliefs (*r* = .44)*	Moderate
			IVI: Three subscales: metaphysical beliefs, positive beliefs and beliefs about loss of control		IUS with BAPS: normalizing beliefs (*r* = .01)	
			PANSS		IUS with BAPS: survival strategy (*r* = .18)	
					IUS with BAPS: total (*r* = .28)	
					IUS with IVI: metaphysical beliefs (*r* = .85)**	
					IUS with IVI: positive beliefs (*r* = .32)	
					IUS with IVI: loss of control (*r* = .71)**	
					IUS with IVI: total (*r* = .82)**	
					IUS with PANSS total: no r (non‐sig)	
Zheng et al. (2022)	Undergraduate students (*N* = 311)	Cross‐sectional	GPTS–social reference and persecution	IUS‐27	IUS and GPTS Social reference (0.57)**	Weak
			PSQ–interpersonal suspiciousness/hostility, mistrust/wariness		IUS and GPTS Persecutory beliefs (0.38)**	
					IUS and PSQ interpersonal suspiciousness/hostility (0.54)**	
					IUS and PSQ mistrust/wariness(0.39)**	

Abbreviations: BAPS, Beliefs About Persecution Scale; CAARMS, Comprehensive Assessment of At‐Risk Mental States; GPTS, Green Paranoid Thoughts Scale; IUS, Intolerance of Uncertainty Scale; IVI, Interpretation of Voices Scale; PANSS, Positive and Negative Syndrome Scale; PDI, Peters Delusion Inventory; PSQ, Paranoia and Suspiciousness Questionnaire; PSYRATS, Psychotic Symptoms Rating Scales; SANS, Scale for the Assessment of Negative Symptoms.

*p < .05.

**p < .001.

## RESULTS

### Summary of studies

A summary of the search process is provided in Figure 1. There were ten studies (reported in nine papers as one paper reports the findings from two studies) that met criteria for inclusion in the review (total *n* = 1503). Preliminary data extracted from studies are presented in Table 1. There were five studies that used a clinical psychosis sample (total *n* = 513), four studies used a nonclinical student sample (total *n* = 932) and one study used individuals with an ‘at‐risk’ mental state (*n* = 35) and matched healthy controls (*n* = 23). Measures of psychosis used across the studies included the following: Scale for the assessment of positive symptoms (SAPS, *k* = 2); Positive and Negative Syndrome Scale (PANSS, *k* = 4); Scale for the assessment of negative symptoms (SANS, *k* = 1) and the Comprehensive Assessment of At‐Risk Mental States (CAARMS, *k* = 1). Measures of individual psychotic symptoms included the following: Psychotic Symptoms Rating Scales (PSYRATS); delusions subscale (*k =* 3); voices subscale (*k* = 1); Green Paranoid Thoughts Scale (GPTS, *k* = 5), Peters Delusion Inventory (*k* = 1); Beliefs about persecution scale (BAPS, *k* = 1); Interpretation of Voices Scale (IVI, *k* = 1) and Paranoia and suspiciousness questionnaire (PSQ, *k* = 1). All studies used the Intolerance of Uncertainty Scale (IUS), including the full 27‐item version (*k* = 4), 12‐item version (*k* = 4), 9‐item version (*k* = 1) and 2‐item version (*k* = 1). There were nine studies that used a cross‐sectional design and one study used a between‐subjects design comparing individuals with an at‐risk mental state and matched healthy controls. In relation to the overall quality of studies, five were rated as moderate and five were rated as weak.

### Main findings

Table one provides a summary of the individual studies included in the review.

#### Intolerance of uncertainty and psychotic symptoms

There were two studies that examined the relationship between intolerance of uncertainty and total positive symptoms (both using the PANSS); one study reported a significant association between intolerance of uncertainty and positive psychotic symptoms with a moderate effect size (Sedighi et al., 2019) and one study found no significant relationship (Lebert et al., 2021). Two studies found a significant relationship between intolerance of uncertainty and negative symptoms, one with a small effect size (Bredemeier et al., 2019) and one with a moderate effect size (Sedighi et al., 2019). One study reported no significant relationship between intolerance of uncertainty and total score on the PANSS (White & Gumley, 2010).

#### Intolerance of uncertainty and delusions

There were two studies that reported a significant association between intolerance of uncertainty and delusions (*r*_range_ = .14, .39), with small–medium effect sizes (Bredemeier et al., 2019; Startup et al., 2016). One study found no significant association between intolerance of uncertainty and delusions (Lebert et al., 2021).

#### Intolerance of uncertainty and hallucinations

There were two studies that reported associations between intolerance of uncertainty and hallucinations, of which one study found no significant association using the SAPS (BBredemeier et al., 2019) and one found a significant association using the IVI with a large effect size (*r =* .82) (White & Gumley, 2010).

#### Intolerance of uncertainty and paranoia

Of note, six studies found significant associations between intolerance of uncertainty and paranoia, with medium‐large effect sizes (*r*_range_ = .38, .72) (King & Dudley, 2017; Larsen et al., 2021 [report findings from two studies]; Lebert et al., 2021; Startup et al., 2016; Zheng et al., 2022). Additionally, a small but nonsignificant effect was reported between intolerance of uncertainty and beliefs about persecution (White & Gumley, 2010), a significant medium effect between intolerance of uncertainty and mistrust and a significant large effect between intolerance of uncertainty and interpersonal suspiciousness/hostility (Zheng et al., 2022).

#### Group differences

There was one study that used a between‐subjects design and found a large effect size (*d* = 1.11) for the difference in intolerance of uncertainty between individuals with an ‘at‐risk’ mental state of psychosis and matched healthy controls (Broome et al., 2007).

## DISCUSSION

The systematic review identified ten studies (published in nine papers) that reported on the relationship between intolerance of uncertainty and psychosis symptoms. Given the small body of evidence, results should be interpreted with caution. Overall, there was no evidence for a relationship between intolerance of uncertainty and total psychotic symptoms (total score on the PANSS), mixed evidence for a relationship between intolerance of uncertainty and positive symptoms (one study reported a significant association and another didn't), and significant associations with small–medium effect sizes reported between intolerance of uncertainty and negative symptoms. Initial evidence, albeit from one study, suggests that intolerance of uncertainty was significantly higher in individuals with an ‘at‐risk’ mental state for psychosis, compared to controls. Notably, there was greater evidence for a relationship between intolerance of uncertainty and individual psychotic symptoms such as delusions and paranoia within clinical and nonclinical samples. More specifically, the majority of studies demonstrated that higher intolerance of uncertainty was significantly associated with more delusions, and all of the studies showed that higher intolerance of uncertainty was significantly associated with more paranoia. Furthermore, there was mixed evidence for a relationship between intolerance of uncertainty and auditory hallucinations in clinical samples, with one study demonstrating that higher intolerance of uncertainty was significantly associated with more auditory hallucinations, while another study reported no significant relationship between intolerance of uncertainty and auditory hallucinations. In general, the findings from the systematic review are in line with prior research suggesting that negative affect (e.g. anxiety and worry; see Freeman et al., 2002; Kuipers et al., 2019; Ludwig et al., 2019) and uncertainty‐related distress in particular (Preti & Cella, 2010) may elicit and maintain psychotic symptoms, including persecutory delusions and auditory hallucinations. Taken together, these findings suggest that intolerance of uncertainty and psychotic symptoms such as paranoia interact at the general population level and that intolerance of uncertainty may be an important transdiagnostic dimension and treatment target for psychotic symptoms such as delusions and paranoia in SSDs. However, this will need to be established through future research.

Overall, the literature is in its infancy, with only ten studies published to date, which include five with clinical populations, four with nonclinical populations and one on individuals with an ‘at‐risk’ mental state, with a range of effect sizes reported across studies. Following publication of additional studies, it will be important in the future to conduct a meta‐analysis to establish the overall summary effect size by individual symptoms and across clinical and nonclinical populations. The tentative results from this systematic review suggest that intolerance of uncertainty may be more strongly related to some individual psychotic symptoms than others. In particular, intolerance of uncertainty was more strongly related to psychotic symptoms such as persecutory delusions and paranoia. The higher order (latent) factor of negative affectivity could be responsible for driving relationships between intolerance of uncertainty and these symptom clusters, as negative affectivity is a shared component that contributes to negative beliefs, emotions (e.g. anxiety) and worry across both anxiety and depression‐related disorders (for review, see Carleton, 2016), and SSDs (Ohi et al., 2016; Van Os & Jones, 2001). Additionally, we observed the findings in relation to intolerance of uncertainty and hallucinations were mixed. It is possible that there was a weaker relationship between intolerance of uncertainty and hallucinations because the latter may be underpinned by several other different lower‐order and higher order (latent) factors. For instance, hallucinations may form on the basis of beliefs, emotions and worry, but may more likely come about through alterations in sensory experience (Allen et al., 2008; Varese et al., 2011). Future research might usefully determine whether there is a relationship between intolerance of uncertainty and beliefs about hallucinations (e.g. what the hallucination might mean and whether the hallucination occurred or not).

Further research is required to examine the specificity of intolerance of uncertainty over other: (1) higher order factors such as negative affectivity (e.g. neuroticism) (for discussion see Morriss, 2023); (2) lower‐order transdiagnostic dimensions associated with anxiety, depression and psychosis such as jumping to conclusions (Bentall et al., 2009); (3) cognitive biases such as need for closure, including use of both existing (e.g. Prisoner's Dilemma Game, see Ellett, Varese, et al., 2023; Ellett, Wildschut, & Chadwick, 2023) and new (e.g. Ambiguous Movie Scene Task, see Hahn et al., 2021) paradigms; (4) common symptoms such as anxiety and depression (Freeman et al., 2012), in predicting and/or maintaining psychotic symptoms such as delusions and paranoia in SSDs. Longitudinal designs will be key for identifying the extent to which intolerance of uncertainty and other known interacting factors (e.g. anxiety, worry; see Freeman et al., 2012; Sun et al., 2018) specifically: (1) predict the onset of psychotic symptoms, (2) maintain psychotic symptoms and (3) can be targeted via treatment (e.g. cognitive behavioural therapy, mindfulness, etc.; see Newman‐Taylor & Bentall, 2023, and Ellett, 2024 for recent reviews) to alleviate psychotic symptoms. Experimental studies using a variety of different task‐based conditions (e.g. uncertainty or paranoia induction) and measurement techniques (e.g. experience sampling methods, action tendencies, psychophysiology) will allow us to ascertain the extent to which intolerance of uncertainty maps onto different facets (e.g. cognitions, affect and behaviour) of psychotic experiences such as delusions and paranoia (for reviews on how this has been achieved for intolerance of uncertainty and anxiety, see Morriss et al., 2023; Tanovic et al., 2018). Applying these efforts across a range of diverse samples and pooling such data through consortiums will be essential for maximizing generalizability and impact (Rodriguez‐Seijas et al., 2023). Ultimately, this work will enable us to understand the positioning and breadth of intolerance of uncertainty within current frameworks (Kotov et al., 2017) and integrative models (Cuthbert, 2022) of transdiagnostic vulnerabilities for SSDs and psychopathology more broadly, providing potentially new lines of enquiry for transdiagnostic treatments.

The systematic review had a few limitations. Firstly, the review identified a small body of literature; thus, caution is warranted when interpreting the findings. In particular, the studies varied in how intolerance of uncertainty (different versions of the same measure) and psychotic symptoms (use of both clinical rating and self‐report measures) were measured, which may explain the heterogeneity in findings for some of the individual psychotic symptoms. Secondly, all of the studies used cross‐sectional designs, making it difficult to infer any causality between intolerance of uncertainty and the onset or maintenance of psychotic symptoms, and research is yet to explore any potential mechanisms. Thirdly, the nonclinical samples consisted of students; therefore, the findings may not generalize to general community samples. Fourth, the overall quality of studies was evenly split, with half rated as moderate and half as weak, which should be taken into account when interpreting the findings of the review. Lastly, the review focused on published literature only, and did not consider grey literature (e.g. unpublished studies), suggesting that there may be publication bias related to positive results.

In sum, despite the small literature, these findings highlight that intolerance of uncertainty may be a relevant risk factor and treatment target for psychotic symptoms such as delusions and paranoia in schizophrenia spectrum disorders. Excitingly, there are several different avenues for future research to explore in order to understand the specificity of the role of intolerance of uncertainty in predicting and maintaining psychotic symptoms in schizophrenia spectrum disorders.

## AUTHOR CONTRIBUTIONS

**Jayne Morriss:** Conceptualization; investigation; writing – original draft; methodology; visualization; writing – review and editing; formal analysis; project administration. **Daisy Butler:** Methodology; project administration; data curation. **Lyn Ellett:** Conceptualization; investigation; writing – original draft; methodology; visualization; writing – review and editing; formal analysis; project administration; supervision.

## CONFLICT OF INTEREST

The authors have no conflicts of interest to declare.

## Data Availability

Data sharing is not applicable to this article as no new data were created or analysed in this study.
